# Charting the therapeutic landscape: a comprehensive evidence map on medical cannabis for health outcomes

**DOI:** 10.3389/fphar.2024.1494492

**Published:** 2024-11-26

**Authors:** Patrícia Montagner, Adán de Salas Quiroga, Arthur Schveitzer Ferreira, Barbara Marinho Duarte da Luz, Bettina Monika Ruppelt, Caio Fabio Schlechta Portella, Carmen Verônica Mendes Abdala, Ricardo Tabach, Ricardo Ghelman, Uwe Blesching, João Paulo Silvério Perfeito, Mariana Cabral Schveitzer

**Affiliations:** ^1^ WeCann Academy, Florianópolis, Brazil; ^2^ Brazilian Academic Consortium for Integrative Health (CABSIN), São Paulo, Brazil; ^3^ Department of Preventive Medicine, Universidade Federal de São Paulo, UNIFESP, São Paulo, Brazil; ^4^ Ginecology Discipline, Department of Ginecology and Obstetrics, Faculty of Medicine, Universidade de São Paulo, USP, São Paulo, Brazil; ^5^ BIREME (Latin American and Caribbean Center on Health Sciences Information), Pan American Health Organization/World Health Organization (PAHO/WHO), São Paulo, Brazil; ^6^ Department of Medicine on Primary Care, Faculty of Medicine, Universidade Federal do Rio de Janeiro, UFRJ, Rio de Janeiro, Brazil; ^7^ Faculty of Science and Therapeutics, Oaksterdam University, Oakland, CA, United States; ^8^ Brazilian Health Regulatory Agency (ANVISA), GMESP, Brasília, Brazil

**Keywords:** medical cannabis, cannabis products, evidence map, CBD, public health, integrative medicine, complementary therapies

## Abstract

The therapeutic potential of medical cannabis has garnered significant attention in recent years, prompting an urgent need for a comprehensive understanding of its effectiveness across various health outcomes. This article presents an Evidence Map that systematically summarizes clinical evidence on the use of medical cannabis, including the health conditions it addresses, the interventions employed, and the resulting clinical outcomes. The objective is to map the effectiveness of medical cannabis in relation to a wide range of health outcomes. The systematic review process involved two independent, blinded literature researchers who screened the search output using Rayyan software. For studies deemed relevant, full texts were obtained to clarify inclusion or exclusion criteria, and any disagreements were resolved through group discussion. Out of 1,840 initial references, 279 potential studies were selected and read in full, resulting in the inclusion of 194 studies in this evidence map. The results highlight the use of various cannabis formulations, including those based on isolated cannabidiol (CBD). Seventy-one distinct health outcomes were identified in the systematic reviews, with the most reported outcomes being related to various types of pain and patient safety. Other frequently studied outcomes included appetite regulation, chemotherapy-induced nausea and vomiting, and muscle spasticity. Notably, 278 out of 489 descriptions of treatment effects for these health outcomes reported either “Positive” or “Potentially Positive” effects. When considering only high-quality systematic reviews, as evaluated by the AMSTAR 2 tool, 42 out of 67 descriptions of treatment effects for up to 20 health outcomes were classified as “Positive” or “Potentially Positive.” These outcomes included pain, insomnia, seizures, anxiety, muscle spasticity, multiple sclerosis, urinary incontinence, anorexia, and patient safety. This evidence map provides a comprehensive overview of the current clinical evidence on medical cannabis, highlighting its potential therapeutic benefits across a range of health conditions and emphasizing the need for further high-quality research.

## 1 Background

The regulation and the wide range of studies among medical cannabis has been changing its role among the therapeutic tools available for practitioners worldwide. Medical cannabis changed over the years from plant-based or extract-based uses, to isolated compounds of plant or synthetic origin in a broad spectrum of vehicles, administration routes, doses and purity (Wyse and Luria, 2021). However, the types, numbers and qualities of the studies have also been dispersed among new medical interventions and health outcomes.

From a pharmacological standpoint, the therapeutic potential of cannabis relies on its main active compounds, namely phytocannabinoids (such as THC and CBD), and partially on other phytochemicals like terpenes and flavonoids (Desaulniers et al., 2021). Although these active compounds elicit biological responses by several mechanisms of action, their ability to interact with cannabinoid receptors and modulate the activity of the endocannabinoid system is at the core of their pharmacological potential (Lu and Mackie, 2021).

It is crucial to conduct thorough studies on medical cannabis, especially with recent investigations such as the University of Colorado’s focus on high-concentration cannabis (Bero et al., 2021) and Groening et al. (2024) evidence mapping on cannabis use and psychosis. The wide variety of product types, conditions treated, and outcomes makes it difficult to draw clear conclusions (Pratt et al., 2019). As data on cannabis use is often mixed, there is an urgent need for high-quality, unbiased, and well-organized evidence. Unfortunately, many studies lack the necessary rigor and objectivity, hindering informed decision-making. Therefore, providing reliable data is essential to support clinical practice and policy development. Hence, the objective of this article is to map the effectiveness of medical cannabis regarding health outcomes.

## 2 Methods

The methodology involved in building an evidence map is similar to that of a systematic review. However, while a systematic review attempts to answer a specific question using a subset of primary clinical studies, an evidence map represents a level above as it is based on systematic reviews generating a broader overview, broadening the horizon of objectives, and has been more widely used to inform public policy.

The Campbell Collaboration suggests that any evidence map is an attempt to create a systematic presentation aiming at organizing information, identifying gaps, facilitating decision-making and visualizing relationships. The map is usually accompanied by a report summarizing the evidence for stakeholders such as researchers, research commissioners, policymakers, and practitioners (Shlonsky et al., 2011).

This Evidence Map summarizes clinical evidence on the use of medical cannabis, including the health conditions, interventions and clinical outcomes. For this evidence map creation, the following six steps were considered (Wyse and Luria, 2021): Definition of the map scope (Desaulniers et al., 2021), Establishment of the working group (Lu and Mackie, 2021), Systematic search and study selection (Bero et al., 2021), Characterization of the evidence (Groening et al., 2024), Generation of the interactive evidence map, and (Pratt et al., 2019) Preparation of the map synthesis. Each step had its own activities involved (Schveitzer et al., 2021). The study was conducted following the International Initiative for Impact Evaluation (3iE) Evidence Gap Methodology, and results were reported according to Preferred Reporting Items for Systematic Reviews and Meta-Analyses (PRISMA) guidelines (Page et al., 2021). The Evidence Maps were supported by a technical expert panel of librarians, practitioners, policymakers, and research experts as collaboration between the Latin American and Caribbean Center on Health Sciences Information, also known as BIREME, is a specialized Unit of the Pan American Health Organization/World Health Organization (PAHO/WHO) and the Brazilian Academic Consortium for Integrative Health (CABSIN).

### 2.1 Data sources

Our search was conducted in BVS, PUBMED and EMBASE databases, from their inception until April 2023, looking for systematic reviews in English, Spanish, and Portuguese. The review question to guide the database search considered the following: (P) general population, (I) use of medical cannabis, (C) placebo and conventional treatments, and (O) health-related outcomes. We consulted experts on this topic and developed the search strategy together with the Latin American and Caribbean Center on Health Sciences Information (BIREME), then entered the query expressions as shown in Supplementary Material.

### 2.2 Inclusion and exclusion criteria

Systematic reviews describing various uses of medical cannabis as clinical intervention for any medical condition with adequate descriptions of health outcomes were eligible for inclusion. We only selected systematic review studies that self-identified as such. Participants of all ages, regardless of health status, were eligible for inclusion if they were under a medical cannabis treatment or trial. We excluded systematic reviews that do not focus on uses of medical cannabis outcomes, systematic reviews using mixed data from medicinal and recreational cannabis use, systematic reviews that do not have at least two sources of primary information for the same intervention or systematic reviews that do not describe medical cannabis use. The complete list of excluded articles and reasons are included as complementary material.

### 2.3 Procedure

Two blinded independent literature researchers screened the systematic review search output through the Rayyan software. When relevant, full texts were obtained to clarify the inclusion/exclusion status. The publications were screened using the inclusion criteria guideline; disagreements were resolved through group discussion.

BIREME, in collaboration with CABSIN, has developed an application for creating evidence maps based on the 3ie methodology, which has already been applied to more than 40 evidence maps. Assessment of Multiple Systematic Review (AMSTAR 2) was applied to analyze the quality (high, moderate, low, and critically low) of the included systematic reviews (Shea et al., 2007). The AMSTAR 2 item quality assessment analysis indicates the degree of confidence in the results of each review and describes the sources of bias: selection, measurement, and confounding factors. From each included systematic review, the type of intervention was extracted, along with the main health outcomes that were summarized across the included studies.

The data about population, treatment effect (positive, potentially positive, inconclusive/mixed findings, no effect, potential negative and negative), estimates for health outcomes, and systematic review characteristics were retrieved.

### 2.4 Data synthesis

We uploaded the data in the Research Electronic Data Capture (REDCap) platform version 14.7.1 to synthesize the findings. For each research paper, the following data was uploaded: Full Title; Publication Journal; Database; Database ID; Publication Date, Publication Country; Focus Country; Full-Text Citation; Interventions; Outcomes Group; Outcomes; Effect; Population (as described in each study); Type of Review; Review Design; Study Design; Quality Assessment; Primary Studies Bibliography. The systematic review outcomes were drafted by one reviewer and discussed by the review team, and the findings were discussed by the review team. We organized the Evidence Map considering the outcomes, effects, and quality assessment of the included systematic reviews. We use the interactive Tableau platform to graphically display all this information.

## 3 Results

**FIGURE 1:**
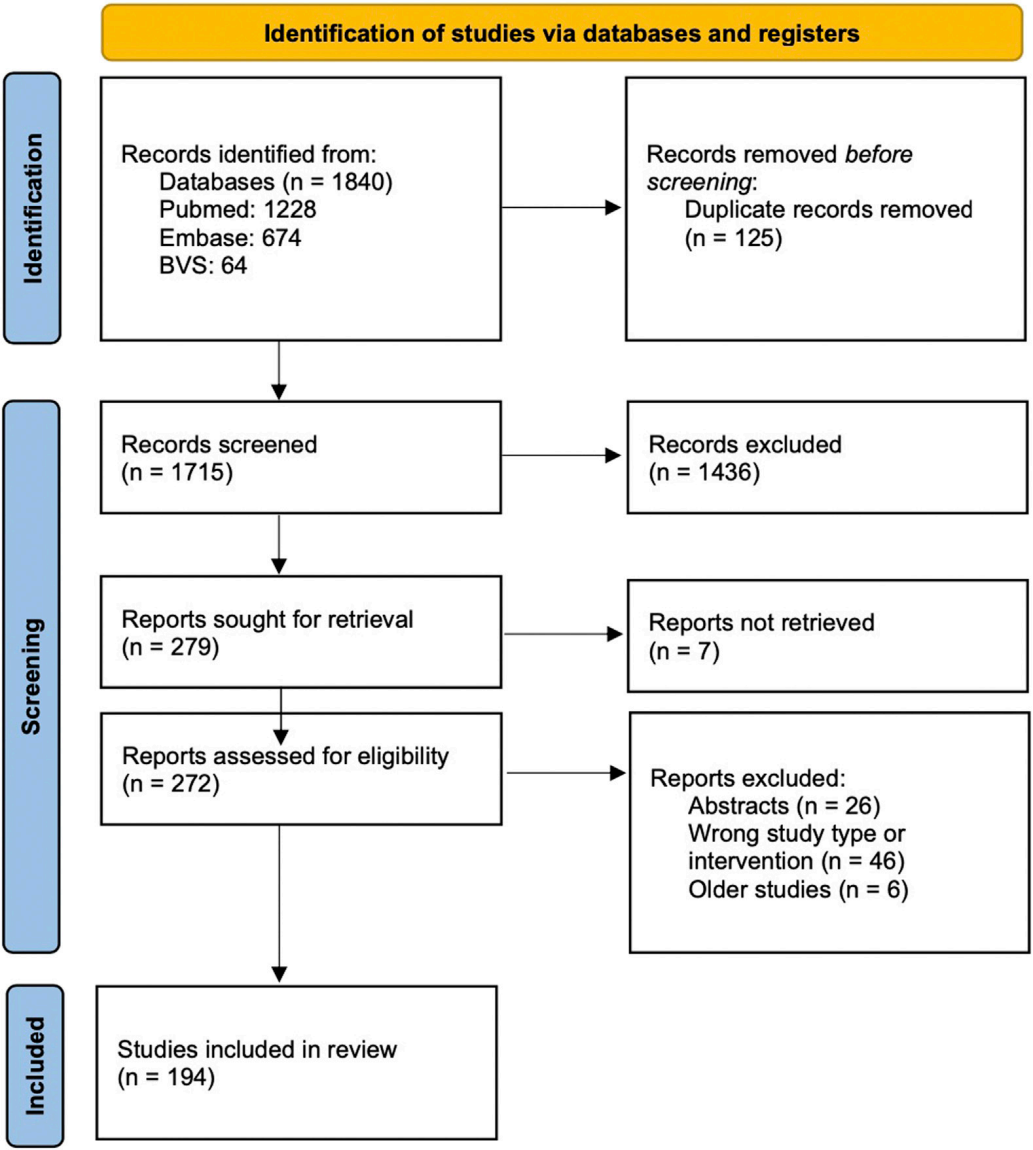
PRISMA Flow Diagram.

From a total of 1840 references, 279 potential studies were selected and read in full, and 194 studies were included in the map. This process is displayed at the PRISMA Flow Diagram (Figure 1).

References could be found from any year, but references that met the inclusion criteria were only found from July 2001 to April 2023. The reasons for excluding studies were: reviews that already have a more recent version (in this case, we only included the most recent version of each review), types of studies that did not meet the inclusion criteria (event summaries, overviews or clinical trials) or studies that evaluated other interventions (did not examine medical cannabis).

The complete list of references of the 194 systematic reviews included in the map is provided in Supplementary Material. The majority of systematic reviews were published from 2018 onwards and more than half of the studies included in the systematic reviews were Randomized Controlled Trials (RCTs).

### 3.1 Interventions

**FIGURE 2:**
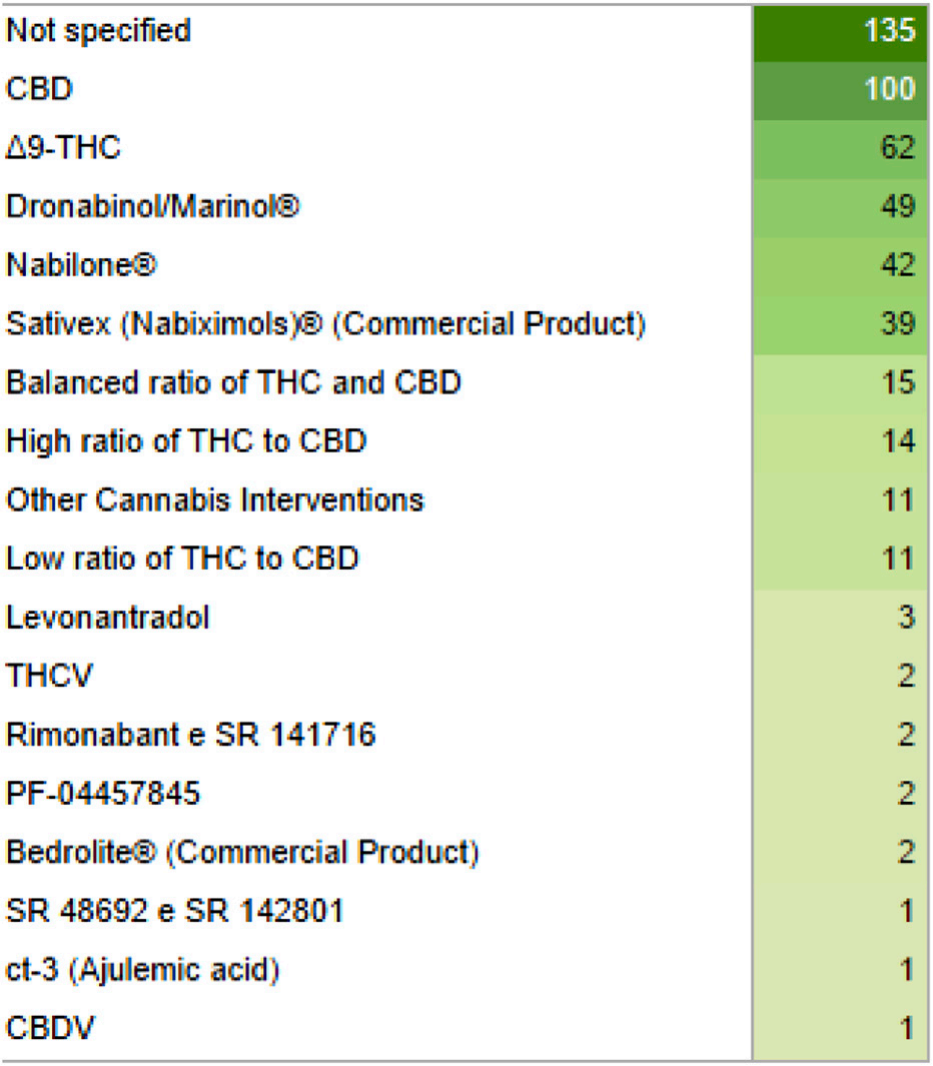
List of interventions and their quantity displayed in green gradient for their quantity, from light to dark green. CBD, Cannabidiol; THC, Tetrahydrocannabinol; THCV, Tetrahydrocannabivarin; CBDV, Cannabidivarin.

The most common interventions found in the studies were varied formulations produced from cannabis and isolated compounds (isolated CBD) (Figure 2).

### 3.2 Population

**FIGURE 3:**
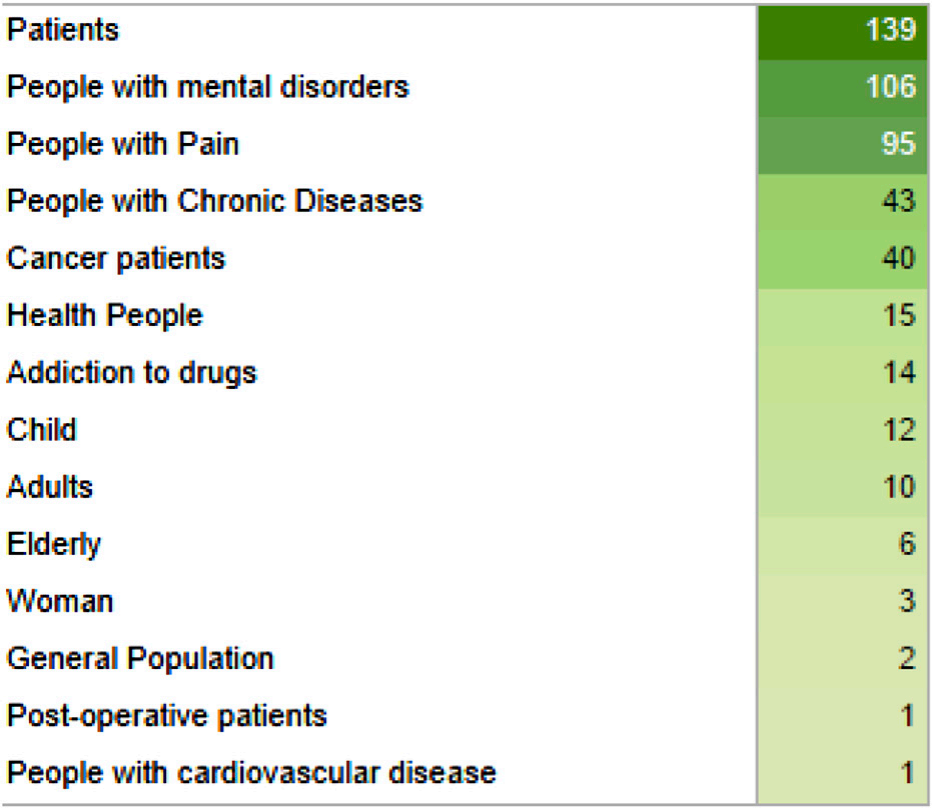
List of studied populations and their quantity displayed in green gradient for their quantity, from light to dark green.

Among the studied populations, four groups had the highest numbers: patients in general, individuals with mental/neurological disorders, individuals with pain, and individuals with chronic disorders (Figure 3).

### 3.3 Countries

Reviews were published in 28 countries, mostly in the United Kingdom (UK) and the United States of America (United States). The main country of focus was the United States, followed by the UK and Canada. The entire list of countries of focus can be found in Supplementary Material.

### 3.4 Health outcomes and treatment effects

**FIGURE 4:**
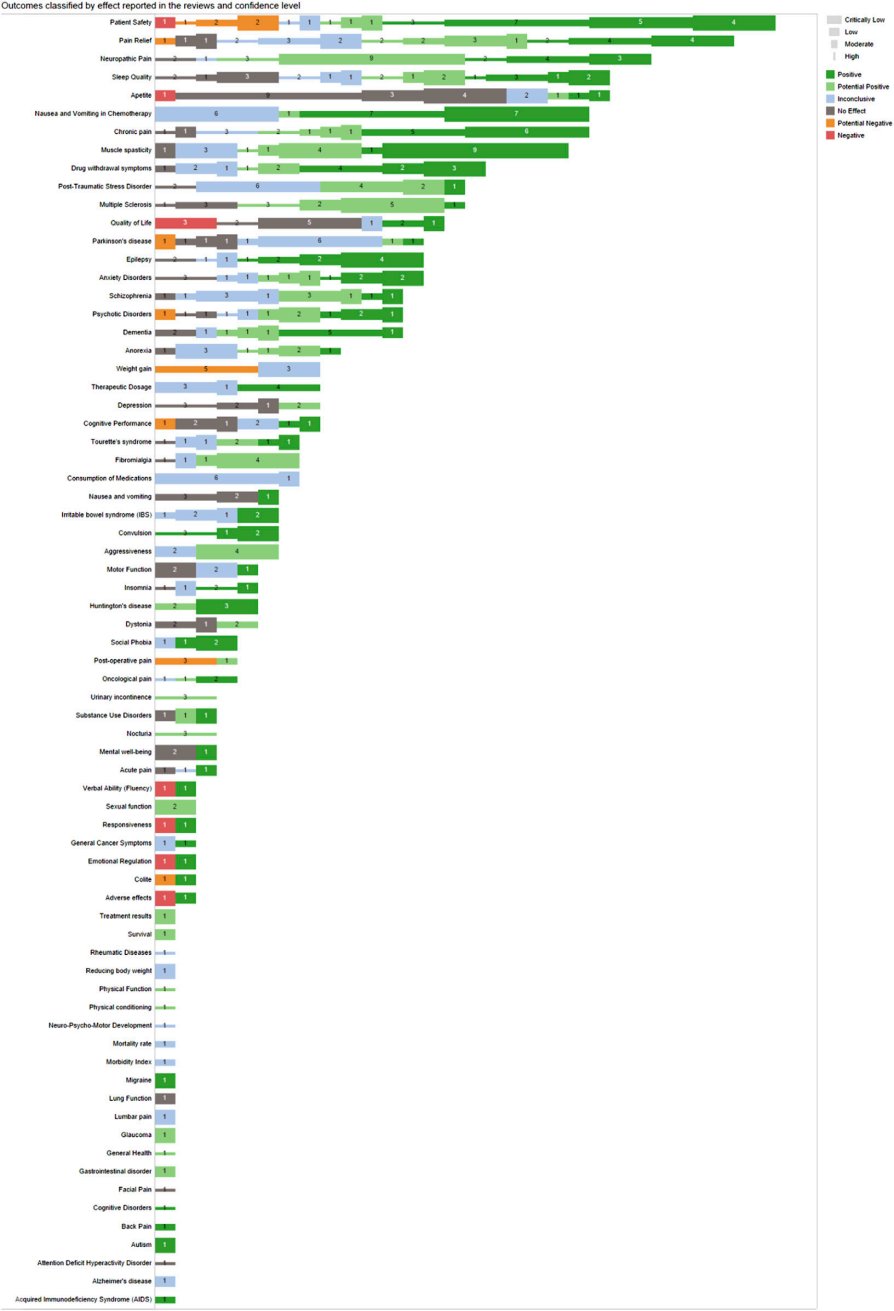
Outcomes classified by effect reported, type of outcome and confidence levels. The types of outcomes are shown from negative to positive, with the thickness of the line indicating the confidence from thinner (more confidence) to thicker (less confidence).

**FIGURE 5:**
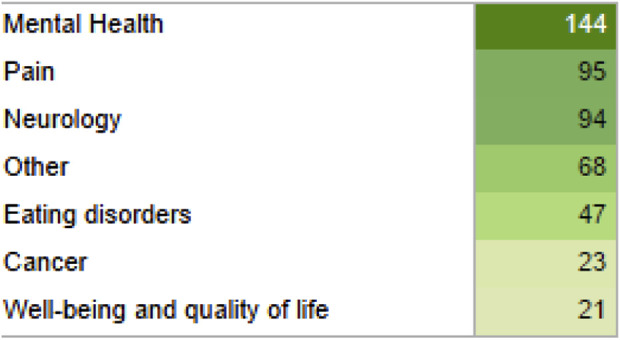
Outcomes classified in seven groups and their quantity displayed in green gradient for their quantity, from light to dark green.

Most health outcomes were related to pain and patient safety. The complete list of the 71 health outcomes analyzed can be found in Figure 4, with its confidence level (critically low, low, moderate and high) and type of outcome (positive, potential positive, inconclusive, no effect, potential negative, and negative effects). The treatment effect preponderant among all health outcomes was “Positive Effects” and top ten most reviewed outcomes: assessing the safety of cannabis-based treatments, pain relief, neuropathic pain, sleep quality, appetite, chemotherapy-induced nausea and vomiting, chronic pain, muscle spasticity, drug withdrawal symptoms and post-traumatic stress disorder. The outcomes were also grouped into seven groups, with the top 3 largest groups of health outcomes being “Mental Health,” “Pain” and “Neurology,” as shown in Figure 5.

**FIGURE 6:**
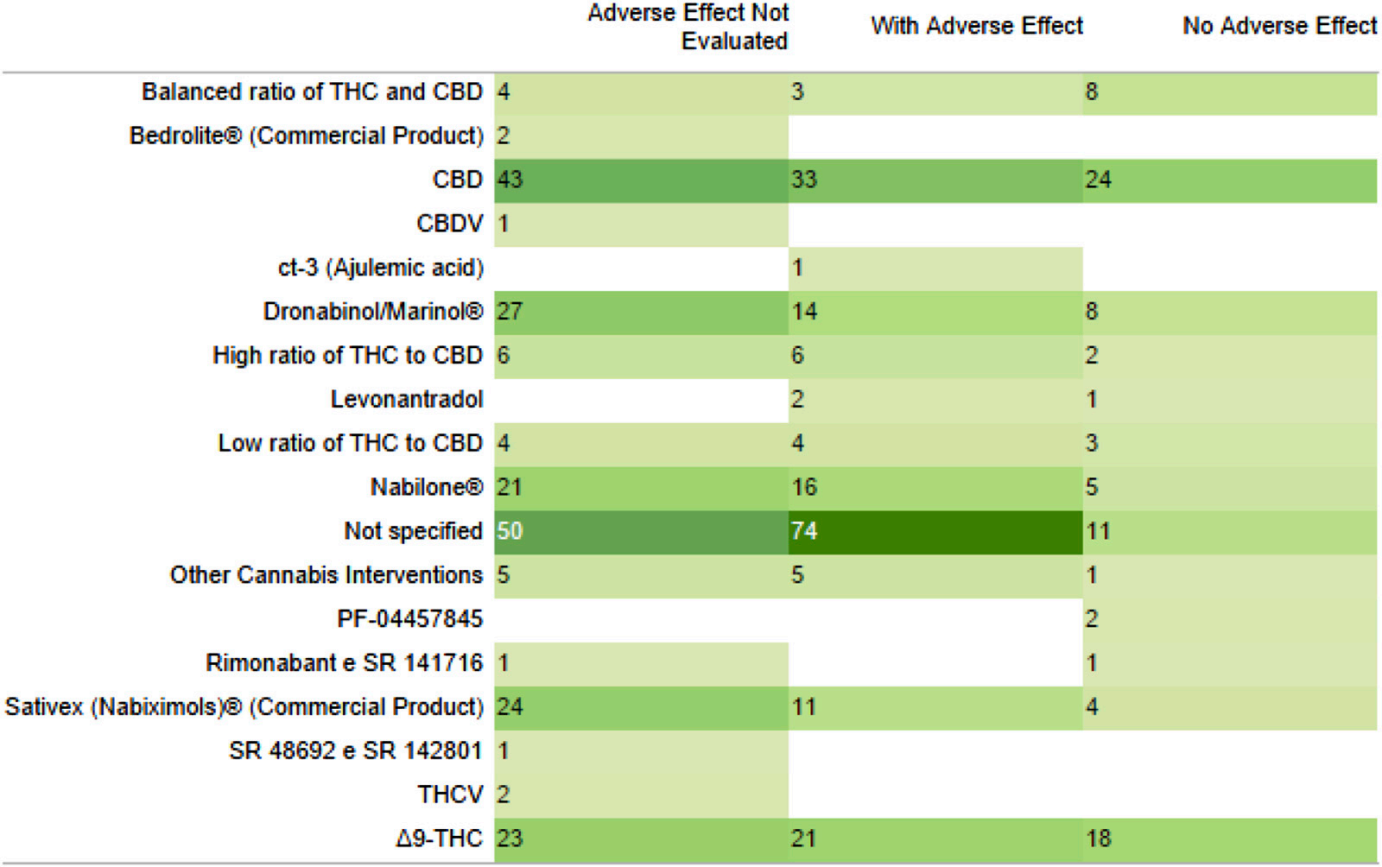
Adverse effects presence, absence or not evaluated with the type of intervention. Their quantity displayed in green gradient for their quantity, from light to dark green.

The majority of reviews also indicated the presence of adverse effects, most of which were mild and tolerable, associated with the interventions, followed by studies that did not evaluate adverse effects and studies that reported no adverse effects associated with the interventions (Figure 6).

**FIGURE 7:**
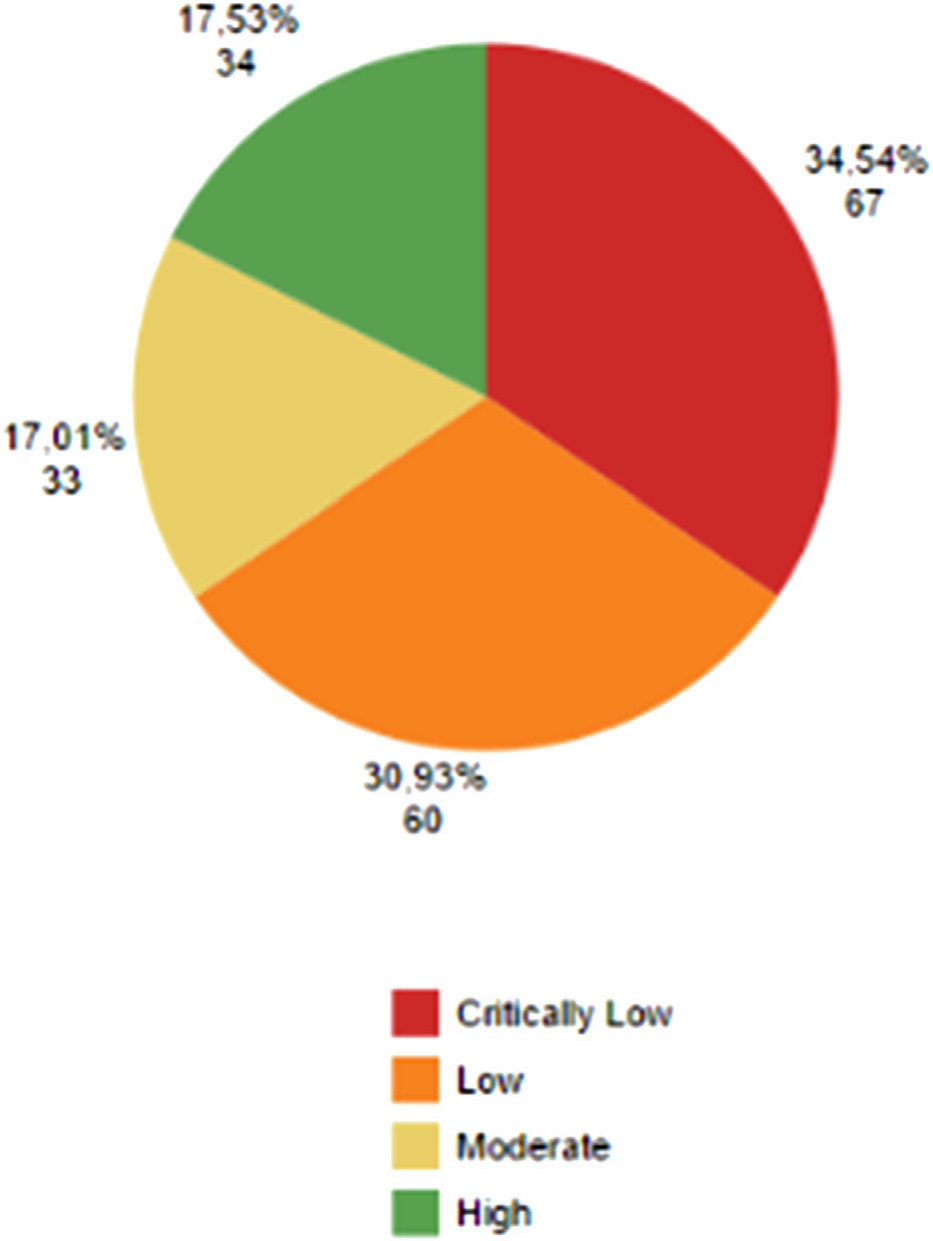
AMSTAR 2 evaluation. In red, critically low confidence studies. In orange, low confidence studies. In light yellow, moderate confidence studies. In green, high confidence studies. Absolute and percentage numbers are displayed near the color area.

The majority of the health outcomes analyzed came from studies of critically low and low quality according to AMSTAR 2 (Figure 7).

### 3.5 High-quality reviews: health outcomes and treatment effects

**FIGURE 8:**
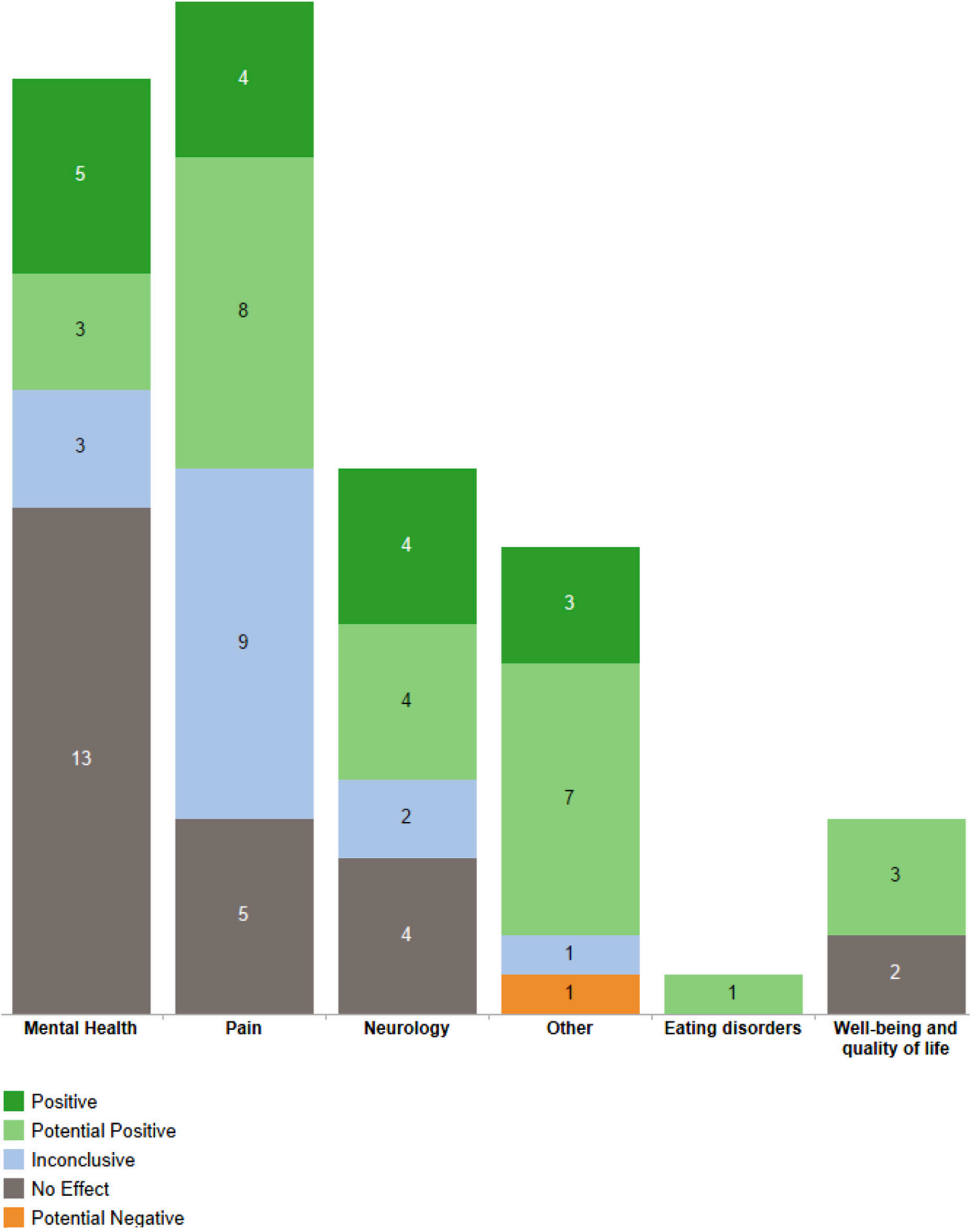
Outcome groups and their types of effects from high-quality systematic reviews. In dark green, positive effects. In light green, potential positive effects. In light blue, inconclusive effects. In dark grey, no effects. In light orange, potential negative effects.

Considering only high-quality systematic reviews, according to AMSTAR 2, there were identified 16 reviews showing “Positive” effects on: patient safety, pain relief, neuropathic pain, insomnia, sleep quality, seizures, epilepsy, anxiety disorders, and cognitive disorders. In addition, 26 systematic reviews showed “Potentially Positive” effects on: chronic pain, cancer pain, muscle spasticity, multiple sclerosis, drug withdrawal symptoms, urinary incontinence, nocturia, anorexia, general health, physical function and physical conditioning, as well as on patient safety, pain relief, neuropathic pain and sleep quality. “Inconclusive” effects were found in 15 systematic reviews for: acute pain, neuropsychomotor development, rheumatic diseases, psychotic disorders and also for patient safety, chronic pain, pain relief, neuropathic pain, cancer pain, epilepsy and quality of sleep. 24 high-quality reviews showed “No Effect” for: depression, post-traumatic stress disorder, Tourette’s syndrome, psychotic disorders, attention deficit hyperactivity disorder, quality of life, facial pain, fibromyalgia and also for anxiety disorders, insomnia, sleep quality, chronic pain, neuropathic pain, multiple sclerosis and epilepsy. Noteworthy, only one high-quality systematic review showed a “Potential Negative” effect on patient safety, when using various cannabis-based and/or unspecified formulations as interventions. Moreover, no high-quality systematic reviews showed a “Negative Effect” on any health outcome. In the seven-group classification of outcomes, no cancer study was high-quality (Figure 8).

### 3.6 Limitations and strengths

A total of 194 studies, 18 cannabis-based medical interventions and 71 health outcomes were identified in the Evidence Map on Medicinal Cannabis. From these studies, 489 interactions between medical interventions, health outcomes and treatment effects were identified. These interactions are listed in Supplementary Material. However, it is worth mentioning that several reviews lack complete data regarding adverse events, dosage and specifying the cannabis product used.

### 3.7 Research gaps

Currently, despite the large number of studies conducted in several countries, there is still a need for more specific studies targeting populations of different ages and clinical conditions (e.g. children, elderly, pregnant women, comorbidities, overweight population, etc.). Also, some pathologies still require larger studies to be conducted (e.g. cardiovascular disorders, kidney diseases, or infections, among others).

## 4 Discussion

Actual evidence-based medicine is the tip of the iceberg among the different medical treatments available for the patient. An evidence map helps the practitioner to guide the therapeutic choices to more safe and efficient treatments. However, it does not fully address the need to tailor treatments to each patient’s unique circumstances, condition, or preferences (Subbiah, 2023).

Over the last six decades, the volume of scientific literature on cannabinoids and the endocannabinoid system (ECS) has grown exponentially. From a mere ten studies in 1962 to an overwhelming 4,428 by 2022, the field has seen a more than four-thousand-fold increase, highlighting an unmatched pace of discovery and investigation (NORML, 2022). As of 2024, approximately 11 new studies are published daily, with more than half bearing potential clinical relevance. This burgeoning body of evidence not only underscores the scientific community’s growing interest in cannabinoid-based therapeutics but also signals a paradigm shift in how we approach and manage chronic conditions.

Despite the existence of several studies and clinical experiences reporting therapeutic benefits using cannabis-based products, most of these studies do not yet present complete information and conclusive and extrapolable evidence on efficacy (European Monitoring Centre for Drugs and Drug Addiction, 2018; Whiting et al., 2015; Brazilian Health Regulatory Agency, 2024). Thus, there are very few cannabis-based products approved worldwide as medicines. Cannabis-based medicines are approved for treatment of seizures associated with two rare and severe forms of epilepsy (Lennox-Gastaut and Dravet syndrome) and tuberous sclerosis complex; and to improve symptoms related to muscle stiffness (spasticity) in multiple sclerosis (Brazilian Health Regulatory Agency, 2024; European Medicines Agency, 2024; Food and Drug Administration, 2024). Considering the gap of complete information on efficacy, in many countries, e.g., Germany, Portugal, Canada and Brazil, laws and regulatory standards that regulates cannabis and cannabis-based products in a specific regulatory category, different from the medicines category, have been approved in order to allow controlled and faster access to them (Brazilian Health Regulatory Agency, 2024; Germany, 2017; Infarmed, 2024; Health Canada, 2024).

The rapid accumulation of knowledge around the ECS and cannabinoid therapeutics presents opportunities and challenges for clinical practice. Despite CBD and Δ-9-THC being the most common defined formulations in this study, many new formulations and synthetic molecules are increasing their importance and clinical relevance. On the one hand, novel insights and emerging treatment trends offer the potential to enhance patient outcomes through more informed and discerning therapeutic decisions (Yang et al., 2021). On the other hand, the sheer volume and pace of new information necessitate a continuous and dynamic process of learning and adaptation for healthcare professionals (Gardiner et al., 2019). Optimizing patient outcomes with cannabinoid-based treatments is an evolving endeavor, one that will grow increasingly effective as evidence quality improves and experiences accumulate.

Beyond the quantitative and qualitative growth of cannabinoid research, a broader cultural shift is affecting the adoption and use of medical cannabis. Independent of legal restrictions, insurance policies, or medical advisories, patients worldwide are increasingly turning to cannabis-based products to alleviate their conditions (Marcu, 2020; Arboleda et al., 2020). This trend is driven by a global zeitgeist favoring patient autonomy and exploring alternative therapeutics, especially when conventional treatments fall short. The significant role of the ECS in modulating various chronic illnesses underscores the importance of understanding and leveraging cannabinoid-based products to achieve precise therapeutic effects, minimize adverse outcomes, and, ultimately, optimize patient care.

The evidence map presented in this study brings forth a comprehensive analysis of 194 systematic review studies on the effectiveness of medical cannabis across various health outcomes. The findings indicate a considerable number of positive and potentially positive effects, from the high-quality studies, particularly in areas such as pain relief, epilepsy, anxiety disorders, insomnia, cancer patients, muscle spasticity, urinary incontinence, anorexia and multiple sclerosis, among others.

Our findings of the most used medical cannabis formulations are in agreement with other scoping reviews interventions (Pantoja-Ruiz et al., 2022; Kirkland et al., 2022), as CBD and Δ-9-THC are the most popular and oldest formulations used. However, scoping reviews focusing in more specific areas and/or populations such as children and adolescents with autism spectrum disorder, for example, may have different majority formulations (Fletcher et al., 2022) due to the particularities of specific groups.

The diversity of outcomes is in accordance with what found in recent literature, likewise as the safety and adverse effects reported. Nonetheless, the qualities of the studies diverge due to the size of the research and the criteria used for classification (Pratt et al., 2019; Montero-Oleas et al., 2020).

While the evidence map offers valuable insights into the efficacy of medical cannabis, it also highlights the need for further high-quality research, particularly from underrepresented regions such as Latin America (Suárez-Jacobo et al., 2023). Enhancing the methodological quality of studies and increasing their number will provide a more robust foundation for clinical decision-making. It is also important to point out the importance to decrease the heterogeneity of formulations and studies in systematic reviews, as well as treat the differences between them more seriously, hence the high number of nonspecific formulations and populations found in this review.

Additionally, as the landscape of cannabinoid therapeutics continues to evolve, there is a critical need for comprehensive education for healthcare providers and patients (Ware and Ziemianski, 2015). Understanding the nuances of the ECS and the therapeutic potential of cannabinoids is essential for navigating the complexities of modern healthcare and ensuring that patients receive safe, effective, and personalized treatment options (Maccarrone et al., 2023).

Specific literature in different areas shows agreement in the heterogeneity of the findings concerning the use of medical cannabis, even in the same pathologies and treatments, due to the mixing of the findings and the quality of the studies (Pratt et al., 2019; Pantoja-Ruiz et al., 2022; Kirkland et al., 2022; Fletcher et al., 2022; Montero-Oleas et al., 2020).

As such, the evidence map on the effectiveness of medical cannabis serves as a crucial step forward in our understanding of cannabinoid-based therapeutics. However, it also underscores the necessity of continued research, education, and adaptation of an ever-expanding and evolving field. As we move forward, the collective goal should be to rationally harness the potential of cannabinoids to improve patient outcomes and advance the practice of healthcare in a rapidly changing world.

## 5 Conclusion

This Evidence Map embodies 194 systematic reviews regarding various uses of medical cannabis. The results highlight the use of various formulations based on cannabis and isolated CBD. Among the 71 health outcomes described in the systematic reviews, the most common were related to various types of pain and patient safety, followed by appetite, chemotherapy-induced nausea and vomiting, and muscle spasticity. Furthermore, 278 out of the 489 descriptions of a treatment effect for any of those 71 health outcomes reported “Positive” or “Potentially Positive” effects. Likewise, when analyses are restricted to AMSTAR 2 high-quality systematic reviews, 42 out of 67 descriptions of treatment effect reported “Positive” or “Potentially Positive” effects for up to 20 health outcomes, pain, insomnia, seizures, anxiety, muscle spasticity, multiple sclerosis, urinary incontinence, anorexia and patient safety, were among the most reported. The results of this map should highlight existing evidence and gaps, the need for additional studies providing confirmatory evidence on effectiveness for some clinical conditions, guide and facilitate decision-making for the clinical community worldwide and support the actions of health-related management for policymakers.
